# The Use of Monensin for Ketosis Prevention in Dairy Cows during the Transition Period: A Systematic Review

**DOI:** 10.3390/ani11071988

**Published:** 2021-07-02

**Authors:** Ludovica M. E. Mammi, Marcello Guadagnini, Gerald Mechor, Juan M. Cainzos, Isa Fusaro, Alberto Palmonari, Andrea Formigoni

**Affiliations:** 1 Department of Veterinary Medical Sciences, University of Bologna, Via Tolara di Sopra 50, 40064 Ozzano Emilia, Italy; alberto.palmonari2@unibo.it (A.P.); andrea.formigoni@unibo.it (A.F.); 2 Elanco Italia Spa, Via dei Colatori 12, 50019 Sesto Fiorentino, Italy; marcello.guadagnini@elancoah.com; 3 Elanco Animal Health, Innovation Way 2500, Greenfield, IN 46140, USA; gerald.mechor@elancoah.com (G.M.); juan_manuel.cainzos@elancoah.com (J.M.C.); 4 Faculty of Veterinary Medicine, University of Teramo, Località Piano D’Accio, 64100 Teramo, Italy; ifusaro@unite.it

**Keywords:** monensin, dairy cow, transition period, cow health, milk quality

## Abstract

**Simple Summary:**

The use of a controlled-release capsule of monensin for the prevention of ketosis in dairy cows, nowadays, has become widespread. In Europe, this is the only use of monensin permitted in dairy cows. Literature regarding the use of this molecule as a feed additive or controlled-release capsule is extensive, and results reported across studies vary with respect to the mode and dose of administration, target animals, and period of lactation. Therefore, the aim of this review was to summarize the literature observations regarding the use of monensin for the prevention of ketosis in dairy cows during the transition period.

**Abstract:**

Since the approval by the European Medicines Agency in 2013 of a monensin controlled-release capsule (CRC) for the prevention of ketosis in dairy cows, there has been widespread use across Europe. In recent decades, several papers have investigated the effects of monensin used as a CRC or as a feed additive to improve cattle energy metabolism and improve feed efficiency. Since the CRC is the only form of monensin permitted in Europe in dairy cows, the objective of this review was to report and summarize observations from the literature on the effects of this treatment in transition cows. The peer-reviewed literature published from 1997 was scanned, and papers written in English were evaluated for eligibility. Only papers evaluating the use of monensin in dairy cows for the prevention of ketosis during the transition period were reviewed. In total, 42 papers met the required criteria and were included in this review. The major findings focused on cow metabolism and health, rumen fermentation and milk production and quality. Overall, the review of the existing literature confirmed that monensin delivered as a CRC during the transition period has effects of different magnitude compared to other forms, doses or durations of administration. Studies agree on the antiketotic effects of this treatment, showing evidence of an increased propionate production in the rumen, reduced blood β-hydroxybutyrate, and improved liver function in treated cows, mainly resulting in reduced incidence of peripartum disease. On the contrary, the effects of CRC on ammonia production and rumen microflora are less robust than those reported for other forms. Of importance for the European market is the well-documented absence of any negative impact on milk and cheese production and composition using the CRC treatment.

## 1. Introduction

Monensin, a carboxylic polyether ionophore, has been used worldwide in livestock as a coccidiostat since its discovery in 1969. The ability of the molecule to selectively reduce Gram-positive bacteria in the rumen, in favor of Gram-negative bacteria, improves efficiency of energy metabolism with several benefits for animals’ health and production [1]. The molecule’s ability to alter rumen fermentation to increase propionate production while decreasing carbon loss in the form of carbon dioxide and methane improves feed efficiency [1,2]. This mode of action has driven the use of monensin for decades as a feed additive in cattle nutrition [2]. Indeed, it was included by the WHO Collaborating Centre for Drug Statistics Methodology (WHOCC), in the list of “Drugs for prevention and/or treatment of acetonemia” (QA16QA06), in addition to the list of “agents against protozoal disease” (QP51AH03) [3].

In Europe, the use of monensin in dairy cows has never been approved, and since the prohibition of growth promotion in 2006, its use for cattle as a feed additive was banned, while it continues to be allowed for the control of coccidiosis in poultry [4].

In 2013, the European Medicines Agency [5] approved the use of monensin in dairy cows as a continuous-release intraruminal device (CRC) for the prevention of ketosis in high-risk cows (Kexxtone, Elanco GmbH, Cuxhaven, Germany). This pharmaceutical form is now the only one permitted in Europe, and it specifically focuses on the transition cow.

The CRC contains 32.4 g of monensin that is released continuously into the rumen throughout a period of 95 d, at a daily dose of 335 mg [5] (EMA, 2013). Therefore, to exert its effects in the prevention of ketosis, it needs to be administered within 3–4 weeks before calving, as indicated on the label (Elanco Animal Health).

Over the last several decades, researchers have published reports on the effects of monensin supplementation on production, health, and ruminal fermentation in dairy cows. 

Administration form, daily dose and target animals used in the published papers vary, as does the magnitude of the responses. Recently, several attempts have been made to summarize scientific results regarding the use of this molecule in dairy cattle, with the publication of exhaustive reviews and a metanalysis [1,6,7,8,9]. Most of these works were conducted in North America, and they include every form of monensin use in dairy cows.

To our knowledge, no papers have been published summarizing results regarding the administration of monensin during the transition period for the prevention of ketosis focusing on the CRC. The objective of this review is to examine peer-reviewed publications on the effects of monensin administration during the prepartum period and the first weeks of lactation, as a tool for ketosis incidence reduction. 

The specific goal of the present work is to elucidate the impact of this treatment on the metabolism and health of dairy cows while examining the consequences on milk production and components.

## 2. Materials and Methods

### Search Strategy and Inclusion Criteria

This review was conducted according to the PRISMA guidelines [10] and analyzed only studies that were peer-reviewed, complete, and written in English.

**Figure 1:**
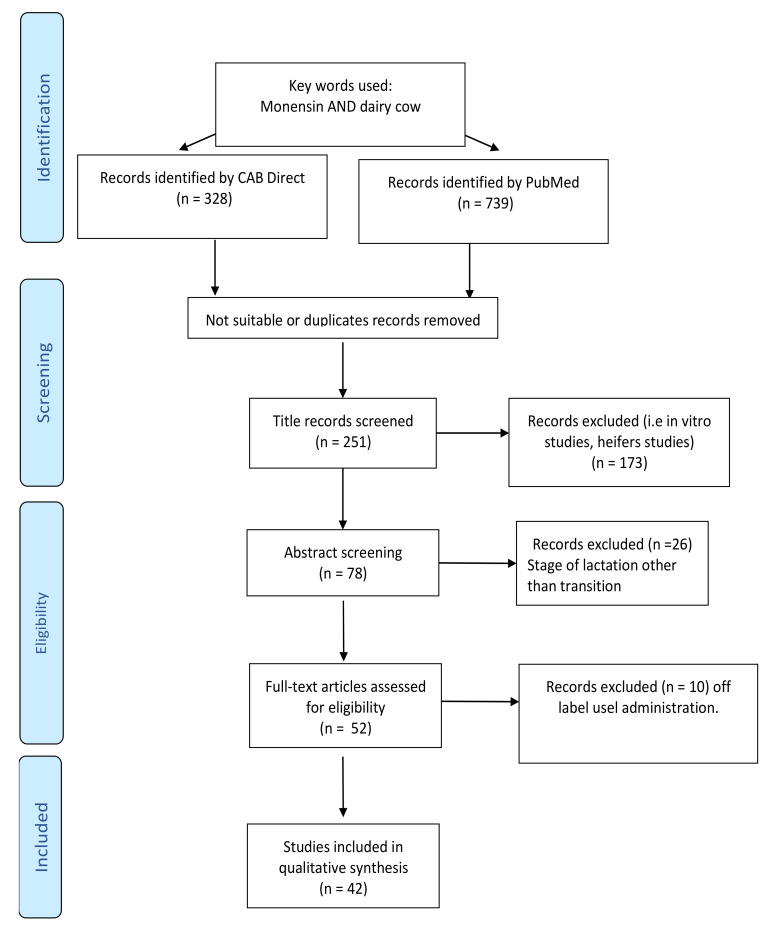
Search strategy and screening process used for the eligibility of the manuscripts according to PRISMA guidelines.

The manuscript screening process is shown in Figure 1. 

In March 2021, the available literature regarding the use of monensin in dairy cows was extracted from the CAB [11] and Pubmed [12] databases using the following key words: monensin AND dairy cow. This search resulted in 739 (Pubmed) and 328 (CAB) studies and included papers published since 1971. However, only papers published after 1996 were evaluated, considering the comprehensive review on ionophores use in dairy cattle, published by Duffield and Bagg in 2000 [1], which analyzed studies published since 1996. 

The first selection was made by scanning the titles of the papers, after which 173 works were excluded because they were outside the scope of the present review (i.e., in vitro or heifer studies). Additionally, abstracts were evaluated and only studies comparing the use of monensin as an antiketoic treatment delivered during the entire transition period were included in the analysis. 

In the majority of these papers, monensin was administered as a CRC; however, 9 studies evaluated the effect of monensin supplemented as a top-dressing. The delivery of a similar daily dose as the CRC (between 300 and 400 mg/d) administered continuously from at least 3 weeks before calving to 60 DIM resulted in the incorporation of these studies as well.

Studies in which CRC was delivered after calving were excluded, as were three studies [13,14,15] in which two capsules were administered, one before calving and one after. Similarly, two studies [16,17] were excluded because CRC was administered between 50 and 70 days before calving, prolonging the time of exposition to the treatment. These uses are not consistent with the approved use of the bolus as indicated on the label.

In summary, 42 studies met the required characteristics and were therefore included in this review.

## 3. Results and Discussion

### 3.1. Rumen Digestion and Fermentation

Monensin exerts its main effects on rumen microbiota by changing the sodium–potassium balance in the cell bacteria, forcing it to spend more energy to restore the balance and causing a reduction in growth and death. This phenomenon affects mainly, but not exclusively, Gram-positive bacteria, and generates a positive advantage over Gram-negative bacteria, causing a shift in ruminal bacteria population [1,2,18].

Among the examined literature, 10 studies evaluated the effects of monensin administration during the transition period on rumen fermentation, digestion, and feed intake (Table 1). 

**Figure 2:**
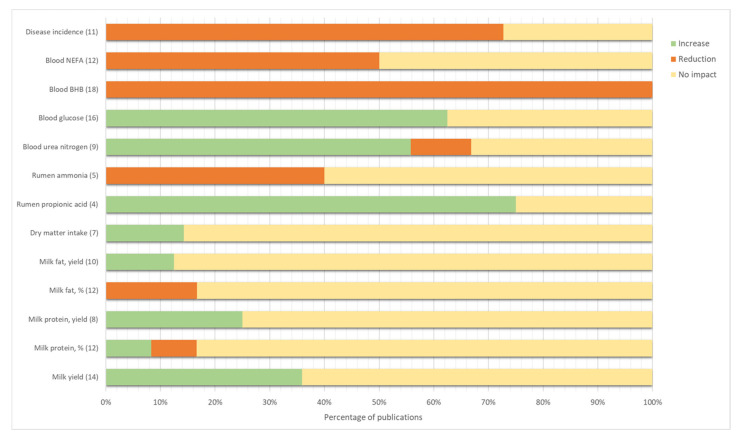
Percentage of publications that demonstrated an impact on a given outcome of monensin administration in early lactation. The effect was considered significant when differences between monensin and control had *p*-values lower than 0.05. No impact corresponds to non-significant differences (*p*-value > 0.05). The number of publications that measured the outcome is given within brackets. BHB = β-hydroxybutyrate; NEFA = non-esterified fatty acids.

Individual dry matter intake (DMI) was recorded in seven of these studies, and most of them (6/7, Figure 2) reported no differences between feed intake of treated and control cows [20,22,23,24,26].

On the contrary, McCarthy et al. (2015b) found an increased DMI in the first 9 weeks after calving, in cows supplemented with monensin during the close-up period (400 mg/d) and the first 60 days of lactation (450 mg/d) [21]. The effect of monensin on DMI has been a debated issue for decades. The increased propionate production driven by monensin influences satiety, thus decreasing DMI in cows that are in positive energy balance [28,29]. On the contrary, results obtained during transition period confirm the absence of this effect in early lactation because propionate production is not at the levels that would drive satiety. For this reason, rumen fill and diet digestibility are considered the most limiting factors for feed intake during the transition period, rather than propionate supply [26,29,30].

Only 6 out of 50 studies analyzed rumen fluid and, according to these reports, total VFA production is not affected by treatment [18,19,20,27]. Additionally, no effects on rumen pH were reported in different studies [19,22,23]. Green and colleagues [27], on the contrary, found increased rumen pH in monensin-treated cows fed a restricted diet (−10% DM) from the 3rd week of lactation, while Plaizier et al. [26] found reduced average time below pH 6 in treated cows. The positive effect on rumen pH could be related to the inhibition of lactate-producing bacteria demonstrated for this molecule [2].

Surprisingly, studies included in this review are not consistent in reporting a clear modification in rumen fermentations pattern, which is, on the other hand, well documented in the literature concerning monensin used as feed additive. The failure to demonstrate significant effects in several studies could be due to the limited number of animals enrolled in transition cow studies, which could reduce the statistical power to demonstrate the impact of the molecule in the ruminal fermentation patterns.

Two studies reported that the treatment increased propionate molar proportion and reduced the acetate:propionate ratio [18,20], confirming results obtained by other in vitro or in vivo experiments with cows in mid or late lactation. Two of these studies evaluated the effect of the treatment in high-conditioned (BCS = 3.95 ± 0.08) cows oversupplied with energy during the dry period to stimulate post-partum lipolysis, and reported significant modifications of propionate and acetate proportion in CRC-treated cows [18,20]. 

The same trend (0.1 < *p* < 0.05) was observed by Green et al. [27] and Mezzetti et al. [19], who additionally reported a significant decrease in valeric, caproic and enanthic acids, suggesting a greater development of cellulolytic bacteria.

Similarly, inconsistent results were found regarding the ammonia content of rumen fluid (Figure 2).

A significant reduction was reported consistently by several of the examined papers [19,26], suggesting a reduced proteolysis in the rumen and a higher proportion of proteins escaped from the rumen in CRC-treated cows that reached the small intestine. The better N balance and the improved N apparent digestibility of treated cows found by Plaizier and colleagues support this theory [26].

However, other authors have reported no variation in rumen ammonia [18,20,27], questioning conclusions about the higher intestinal availability of proteins. 

A unique study investigated the effect of CRC treatment also on immunological parameters of rumen fluid [19]. These authors found a reduced infiltration of T and B lymphocytes in treated cows, suggesting a stabilization of the rumen environment. However, this aspect remains mostly unexplored.

Limited studies have evaluated rumen microbiota. Only Schären et al. [18] and Drong et al. [20] reported on the effect on rumen microbiota, showing no effects on protozoa and archaea populations.

Actually, Schären and colleagues highlighted a reduced bacteria diversity in CRC-treated cows, with a significant prevalence of propionate and succinate producers. In particular, these authors related rumen microbe’s resistance to monensin to the constitution and thickness of their cell wall, more than a clear distinction between gram positive and negative bacteria.

This adaptive characteristic expressed by some bacteria after monensin exposition is a phenotypically expressed trait, and it was shown to be reversible in the absence of monensin within a few generations [31]. 

This aspect is particularly important when considering the issue of antimicrobial resistance. Indeed, recently, the European Medicine Agency [32] stated that, presently, cross-resistance with therapeutical antibiotics and transferable resistance have not been identified and conclude that the use of monensin in animals has no negative impact on public health [32,33].

### 3.2. Metabolism and Health

Twenty-seven papers investigating the effects of monensin treatment during the transition period on cows’ metabolism and health have been included in the present review (Table 2).

The majority of these works focus their attention on the energy balance of the animals, given that this is the main target of the treatment and one of the main challenges in dairy cows after calving.

In particular, several researchers have investigated the effect of the treatment on the blood levels of ketone bodies, NEFA (Non-Esterified Fatty Acids) and glucose (Figure 2).

Overall, most of the studies are consistent in reporting a better energy balance of cows treated with CRC compared to control animals. In particular, results report strong evidence of the reduction of β-hydroxybutyrate (BHB) in treated cows. Indeed, all the analyzed papers report significant lower levels of ketone bodies in the post-partum period, and in several studies even before calving, suggesting a better energy balance of treated cows also before parturition [36,46]. 

Conversely, the effect of monensin CRC on NEFA and glucose levels is less robust. As also confirmed by a recent paper [34], lower levels of NEFA and higher levels of glucose are frequently reported in CRC-treated cows. On the contrary, other studies report no significant effects on these variables. However, no studies demonstrate increased NEFA or lower glucose. This aspect is of particular importance, because overall they confirm the benefit of the treatment in the prevention of ketosis in high-risk cows. Indeed, in a very comprehensive meta-analysis, Duffield and colleagues show that, while the effect of monensin on BHB and acetoacetate is strong and clear, the response of glucose and NEFA levels is consistent but not always statistically significant. Despite studies demonstrating a uniform response, the lack of significant results in some papers is due to the large sample size needed to highlight these differences, which is difficult to achieve in studies with transition cows [6].

The same observations are made relative to loss of body condition.

Three studies enrolling 1010, 136 and 100 cows, respectively [24,34,50] highlighted a reduced loss of body condition in treated animals after calving, which was associated with their lower BHB levels and confirm their better energy balance. On the contrary, another study, with 168 cows, did not find a significant difference in body condition, despite a lower production of ketone bodies of treated cows [40].

Another interesting response to the monensin CRC treatment is the increased plasma level of urea [25,35,46,50] This result was observed by several authors, and it was attributed to an increased flux of undegraded proteins to the intestine, escaping the rumen due to the reduction of protein deaminating bacteria, and the subsequent use of absorbed AA for gluconeogenesis [50]. This explanation was also supported by the higher urea content of milk, reported by several authors [21,22], and by the lower levels of ammonia in the rumen fluid of CRC-treated cows reported, by various authors [19,26]. Another proposed hypothesis is that urea synthesis in the liver increases during the treatment because of the lower lipid accumulation in the hepatocytes that consequently improve its functionality [25]. Indeed, Zahra and colleagues reported a tendency for lower TG in CRC-treated cows at 3 weeks after calving, while McCarthy and colleagues reported lower TG content in multiparous treated cows [21,25]. Conversely, Mullins [22] and Fiore [34] did not find any differences between TG content of treated and control cows. However, Mullins and colleagues reported higher liver mRNA abundance of carnitine-palmitoyl transferase 1a, which is responsible of the translocation of fatty acids from cytosol to mitochondria, thus improving fatty acids oxidation. Additionally, these results were associated with a slow rate of TG accumulation in the liver in the first week after calving [22] and to a better conversion rate from propionate to glucose of CRC-treated cows [21].

The hypothesis of a better liver functionality is also supported by the lower activity of aspartate-aminotransferase (AST) and higher levels of cholesterol in CRC-treated cows [6,20,25,46,50]. On the contrary a lack of response on insulin secretion is reported consistently in the reviewed literature [6,22,24,51] 

As a consequence, the positive effects of monensin CRC treatment on energy metabolism results into lower incidence of post-partum diseases consistently reported in literature.

In particular, treated animals have a lower risk of clinical and subclinical ketosis, displaced abomasum and mastitis [8,39,40,49] A reduced incidence of metritis in CRC-treated cows was also reported in one large study [8,43] that enrolled more than 2000 cows. Results on this outcome are not consistent among other studies [19,22], largely as a function of the large samples size needed to detect a significant effect on disease incidence [8]. 

The mechanism suggested for this reduction is related to the better immune function of treated animals, caused by the improved energy metabolism [8]. In particular, neutrophils and lymphocytes activities are known to be impaired by elevated BHB and NEFA levels [52,53]. However, this aspect was evaluated by only two studies [38,54], which did not find remarkable improvements in the innate immune system in cows treated with monensin. However, Yasui and colleagues [38] reported a tendency for a better function of neutrophils and monocytes in cows fed 450 mg/d of monensin during the transition period, suggesting a beneficial effect of the treatment on these leukocytes. 

The effects of monensin CRC treatment on inflammatory markers such as acute phase proteins is inconsistent [19,21]. Very few studies have investigated inflammatory markers in CRC-treated cows [19,22,44,54] In a large study involving 1010 cows, Crawford and colleagues found increased haptoglobin concentration in unhealthy CRC-treated cows and a lower concentration in healthy animals. On the contrary, no significant difference was found in this protein by other researchers [19,22,54] in considerably smaller studies. Aside from circulating acute phase proteins, Drong and colleagues measured the abundance of mRNA encoding for these proteins in the liver of treated and control cows fed a high concentrate diet before calving [54]. Results obtained by this researcher failed to demonstrate a clear difference in treated animals compared to controls. However, in treated cows, the increase of liver genes expression of haptoglobin and glutathione peroxidase 3 at day 7 after calving compared to pre-calving samples was the most prominent. These results suggest a rapid activation of antioxidant mechanisms in these animals, but a clear association between these variables and the lower production of ketone bodies was not established [54]. 

Despite the positive effect of the treatment on health disorders, however, results regarding a reduction of culling risk after calving are lacking. Only one study evaluated this outcome, reporting a significant reduced culling rate for treated cows in the first 94 days of lactation [55]. As stated by the same author in a meta-analysis [8], to measure the effects on culling rate and disease incidence, a huge sample size is needed, which is very difficult to achieve in studies with transition cows.

Interestingly, in two recent papers, Raboissons and colleagues [56,57] calculated the impact of a systematic reduction of subclinical ketosis in dairy farms on the use of antimicrobials [56] and the economic impact of the CRC treatment [56,57]. With the use of a stochastic model, they calculated a reduction in the use of antimicrobials varying from the 11 to 25%, depending on the levels of reduction of the prevalence of ketosis [56]. In another work, the same authors evaluated the cost effectiveness of the CRC treatment in the prevention of ketosis. The authors calculated that the treatment of the entire herd without a proper strategy to reduce the risk of ketosis is not profitable in the long term, whereas providing an adequate nutrition plan during dry period and the treatment of only cows at high risk is the most effective and convenient strategy to reduce the incidence of ketosis [57]. 

Similarly, Gohary and colleagues [58] calculated a positive return on investment of CRC treatment, when the economic impact of peripartum disease and the increased milk production of treated cows were considered. The impact of the investment depends mainly on the cost of the treatment, the incidence of ketosis, and the percentage of fat cows in the herd [58]. 

### 3.3. Milk Production and Characteristics

For decades, there have been inconsistencies regarding the effect of monensin on milk production and composition.

Most published papers analyzed its effects when administered as a feed additive, while, to the best of our knowledge, only 15 studies evaluated the effects of the CRC treatment on these outcomes (Table 3).

Overall, the review of these studies highlighted contrasting results, with most of them reporting no significant effect of this treatment on milk yield, fat and protein content (Figure 2). On the contrary, few studies showed increased milk production in treated cows [40,48,60] or in treated cows with retained fetal membrane [43]. Similarly, Duffield and colleagues [7] highlighted a positive effect of monensin on milk yield, even though the effect was more marked in the top-dressing form.

Arieli et al. [40] found increased milk production at days 3 and 5 post-partum, and McCarthy et al. [60] in the first 9 weeks of lactation. Similarly, Duffield and colleagues found a higher milk yield for treated cows with BCS at calving greater than 3.25, and particularly in those with BCS higher than 4, while no differences were found among thin cows [48]. Interestingly, Melendez and colleagues reported a higher milk production of treated cows, among those with RFM [17].

These results suggest a larger effect of the treatment on milk production in periods of high-energy demand and, particularly, for cows at higher risk of ketosis, such as early fresh, fat or sick animals. During disease, the inflammatory response increases the negative energy balance of fresh cows; it reduces feed intake, dramatically increases energy requirements and provoke a general reallocation of nutrients from production and growth towards the immune system [62]. 

Regarding milk composition, most of the examined papers report no effect of the CRC treatment on milk components. Indeed, in a metanalysis, Duffield and colleagues report a significant influence of delivery method on milk component percentage and yield, highlighting a lower effect of the CRC form, for both fat and protein yield [7]. Phipps et al. [61] demonstrated a lower percentage of milk fat in CRC-treated cows’ milk, while other research with the CRC reported no differences in fat yield. Conversely, Mezzetti and colleagues [19] reported a lower fat yield in primiparous (PR) treated cows only, suggesting a different response in primiparous and multiparous cows. The same treatment–parity interaction was found by these authors in the protein content, with higher results in treated PR cows [19]. 

This finding is in partial agreement with the meta-analysis of Duffield et al. [7], who reported a heterogeneous effect of monensin on protein content, with several papers reporting a lower protein percentage in treated cows, but a higher protein yield. However, the literature regarding the CRC treatment examined in the present work consistently reports no effect on neither milk protein percentage nor milk protein yield (Figure 2). Only two studies [7,61] reported lower protein percentage in treated cows milk, but, similarly to other studies, no differences in protein yield. The results are in agreement with Duffield et al. [7], who reported a lower effect on milk component of the CRC form compared to the top dressing. This difference could also be explained by the stage of lactation of CRC-treated cows, and the relative negative protein balance typical of transition period [26]. Contrasting results are observed with respect to MUN, with two studies reporting increased levels in treated cows [22,60], and four studies reporting no differences. As previously explained, the suggested mechanisms beyond this result could be the improved liver functions of treated cows [6] and the ruminal protein sparing effect of monensin [22]. 

No studies, to our knowledge, have evaluated the effects of monensin CRC treatment on the fatty acids profile of milk. Only one study evaluated the fatty acids profiles of cheese produced with treated and untreated cows’ milk [59]. The results reported by this study were consistent with those of studies analyzing the fatty acid content of milk produced by cows treated with monensin delivered as a feed additive [7], and show a reduction in the content of short and medium-chain fatty acids and an increased in the long chain ones, particularly C18:1. This effect is associated with the change in the biohydrogenation process caused by the altered rumen microflora. In diets with high content of unsaturated fat, this response is related to the decrease of milk fat. Indeed, this relation was not reported in the study of Mammi et al., in which cows were fed a diet with low levels of unsaturated fat, typical of Parmigiano Reggiano diets [59].

In this latest study, the authors also evaluated cheesemaking properties of milk (coagulation time and curd characteristics) and the quality and organoleptic profile of the Parmigiano Reggiano cheese, reporting no effects of the treatment. Conversely, the only other study that evaluated the cheesemaking properties of milk after CRC treatment showed an impairment of coagulation time and curd firmness in primiparous treated cows [19], but not in multiparous. However, the analysis of the composition and quality of long-ripened cheese confirms that CRC monensin treatment does not affect cheese production or quality [59].

## 4. Conclusions

The present work examined the existing literature focused on the use of monensin during transition period of dairy cows in order to summarize the effects of this treatment on cows’ metabolism and health, rumen fermentation and milk production.

Most papers published in the last two decades examined the effects of the treatment on animals’ metabolism and health. The results reported in this area are more consistent and stronger than those concerning other aspects. The major findings confirm the antiketotic effects of monensin when administered during the transition period, showing evidence of improved energy status and liver function in treated cows that mainly results in reduced incidence of peripartum disease.

Papers that analyzed rumen fermentation consistently reported an increase in propionate production in transition treated cows, while effects on ammonia production and rumen microflora are less evident that those reported in monensin delivered as feed additive. 

The effects of this treatment on nitrogen use have not been completely elucidated and deserve further investigations to clearly establish the role of this treatment on nitrogen balance, which is of particular importance for dairy cows during the transition period. Similarly, little has been reported on the impact of the CRC treatment on cow longevity and on the use of drugs in dairy farms. These issues could be of great interest, considering the impact of this treatment on the reduction of peripartum disease and the European commitment in in increasing animal welfare while reducing antimicrobial treatments in animal productions.

Conversely, the absence of negative impact on milk production and composition for CRC treatment is well documented, for both milk and cheese production. This aspect is particularly important for geographical areas in which great concern exists about the quality and manufacturing properties of milk produced by supplemented cows.

Overall, the review of the existing literature confirms that monensin delivered as a CRC during the transition period has different effects from other forms of administration.

## Figures and Tables

**Table 1:** Studies evaluating the effect of monensin during transition period on feed intake and rumen environment.

Study	Cows, n	Dose ^1^	Delivery Method	Outcomes Measured	References
Mezzetti et al., 2019	38	335	CRC ^2^	Rumen fluid: pH, ammonia, VFA ^3^, immunoglobulins, lymphocytes, myeloid cells	[19]
Schären et al., 2017	60	335	CRC	Rumen fluid: LPS, ammonia, VFA, microbiome characterization	[18]
Drong et al., 2016	60	335	CRC	Individual DMI, rumen pH, ammonia, VFA, protozoa count	[20]
McCarthy et al., 2015b	70	400 pre partum 450 post partum	Top-dressing	Individual DMI, total dry matter, NDF and starch digestibility	[21]
Mullins et al., 2012	32	400 pre partum 450 post partum	Top-dressing	Individual DMI, meal frequency, rumen pH	[22]
Fairfield et al., 2007	16	335	CRC	Individual DMI, pH.	[23]
Petersson-Wolfe et al., 2007	136	335	CRC	Individual DMI	[24]
Zahra et al., 2006	182	335	CRC	Individual DMI	[25]
Plaizier et al., 2000	16	335	CRC	Individual DMI, NDF digestibility, rumen ammonia content	[26]
Green et al., 1999	52	335	CRC	Rumen ph, ammonia, VFA	[27]

^1^ Daily dose (mg/day); ^2^ controlled-release capsule; ^3^ volatile fatty acids.

**Table 2:** Studies evaluating the effect of monensin during transition period on metabolism and health.

Study	Cows, n	Dose ^1^	Delivery Method	Outcomes Measured	References
Fiore et al., 2021	100	335	CRC ^2^	Blood NEFA, BHB, glucose, urea, AST, ALT, GGT, TG ^3^, CHL ^4^, bilirubin, total protein, albumins, globulins, BCS.	[34]
Kasap et al., 2020	50	335	CRC	Blood NEFA, BHB, glucose and urea	[35]
Mezzetti et al., 2019	38	335	CRC	Blood NEFA, BHB, acute phase proteins, reactive oxygen species and disease incidence	[19]
Hausmann et al., 2018	58	336	CRC	Blood NEFA, BHB, glucose, urea, CHL, TG and bilirubin	[36]
Markantonatos et al., 2017	8	300	top-dressing	Glucose kinetic parameters	[37]
Drong et al., 2016	60	335	CRC	Blood NEFA, BHB, glucose and liver total lipids	[20]
Yasui et al., 2016	70	400 pre partum 450 post partum	top-dressing	Blood immune functions	[38]
Compton et al., 2015	837	335	CRC	Blood BHB	[39]
McCarthy et al., 2015	70	400 pre partum 450 post partum	top-dressing	Blood NEFA, BHB and glucose, liver TG and oxidative capacity	[21]
Mullins et al., 2012	32	400 pre partum 450 post partum	top-dressing	Blood NEFA, BHB, glucose, insulin, haptoglobin and liver function.	[22]
Arieli, et al., 2008	168	335	CRC	Blood NEFA, BHB, Glucose, AST and BCS	[40]
Duffield et al., 2008 a, c	Meta-analyses			Blood NEFA, BHB, glucose, urea, CHL, insulin, calcium, phosphorus, MUN	[6,8]
McDougal et al., 2008	772	335	CRC	Incidence of mastitis	[41]
Peterson-Wolfe et al., 2007	136	335	CRC	Blood NEFA, BHB, glucose, insulin, urea, AST, bilirubin, cortisol and BCS	[24]
Kennermann et al., 2006	40	335	CRC	Blood NEFA, BHB, glucose, urea, TG	[42]
Melendez et al., 2006b	2025	335	CRC	Incidence of retained fetal membranes and reproductive performance	[43]
Zahra et al., 2006	182	335	CRC	Blood BHB, glucose, AST, ALT, urea	[25]
Crawford et al., 2005	1010	335	CRC	Serum haptoglobin	[44]
Plaizier et al., 2005	16	335	CRC	Blood NEFA, BHB, glucose and urea	[45]
Duffield et al., 2003	251	335	CRC	Blood NEFA, BHB, glucose, urea, CHL, calcium, phosphorus and BCS	[46]
Duffield et al., 2002	1317	335	CRC	Disease incidence	[47]
Duffield et al., 1999a	1010	335	CRC	Disease incidence, culling rate, reproductive performance	[48]
Duffield et al., 1998a, b	1010	335	CRC	Blood and milk BHB, glucose, AST, urea, total protein, calcium, phosphorus and BCS	[49,50]
Green et al., 1999	52	335	CRC	Blood BHB, glucose and BCS	[27]
Van der Werf et al., 1998	58	300	top-dressing	Blood BHB, glucose, acetoacetate, insulin	[51]

^1^ Daily dose (mg/day); ^2^ controlled-release capsule; ^3^ triglycerides; ^4^ cholesterol.

**Table 3:** Studies included in this review evaluating the effect of monensin during transition period on production performance.

Study	Cows, n	Dose ^1^	Delivery Method	Outcomes Measured	References
Kasap et al., 2020	50	335	CRC ^2^	MY ^3^	[35]
Mezzetti et al., 2019	38	335	CRC	MY, fat % and yield, protein % and yield, casein %, lactose %, urea, SCC, titrable acidity and cheesemaking properties	[19]
Hausmann et al., 2018	58	336	CRC	MY, ECM, Fat % and yield, protein % and yield, urea, SCC	[36]
Mammi et al., 2018	91	335	CRC	Fat %, protein %, casein %, lactose %, urea, SCC, titrable acidity, pH, total bacteria count and cheesemaking properties. Whey starter fermentative activity, cheese yield, composition, sensory profile and fatty acids.	[59]
McCarthy et al., 2015a	70	400 pre partum 450 post partum	top-dressing	MY, ECM, FCM, fat % and yield, protein % and yield, lactose % and yield, urea, SCS	[60]
Mullins et al., 2012	32	400 pre partum 450 post partum	top-dressing	MY, fat %, protein %, lactose %, urea	[22]
Arieli, et al., 2008	168	335	CRC	MY, fat % and yield, protein % and yield, lactose % and yield, urea, SCC	[40]
Duffield et al., 2008b	Meta-analysis			MY, fat % and yield, protein % and yield	[7]
Fairfield et al., 2007	16	335	CRC	MY, fat % and yield, protein % and yield, lactose %	[23]
Melendez et al., 2006b	2025	335	CRC	MY	[43]
Zahra et al., 2006	182	335	CRC	MY, fat %, protein %	[25]
Plaizier et al., 2000	16	335	CRC	Milk nitrogen	[26]
Phipps et al., 2000 ^4^	98	300	top-dressing	MY, fat % and yield, protein % and yield	[61]
Duffield et al., 1999b	1010	335	CRC	MY, fat %, protein %	[48]
Van der Werf et al., 1998	58	300	top-dressing	MY, fat % and yield, protein % and yield	[51]

^1^ Daily dose (mg/day); ^2^ controlled-release capsule; ^3^ milk yield; ^4^ 2nd lactation of experiment 2.
